# Monitoring Network Confirms Land Use Change is a Substantial Component of the Forest Carbon Sink in the eastern United States

**DOI:** 10.1038/srep17028

**Published:** 2015-12-07

**Authors:** C. W. Woodall, B. F. Walters, J. W. Coulston, A. W. D’Amato, G. M. Domke, M. B. Russell, P. A. Sowers

**Affiliations:** 1 USDA Forest Service, Northern Research Station, Forest Inventory and Analysis Program, St. Paul, MN, USA; 2 USDA Forest Service, Southern Research Station, Forest Inventory and Analysis Program, Blacksburg, VA, USA; 3 University of Vermont, Rubenstein School of Environment and Natural Resources, Burlington, VT, USA; 4 University of Minnesota, Department of Forest Resources, St. Paul, MN, USA

## Abstract

Quantifying forest carbon (C) stocks and stock change within a matrix of land use (LU) and LU change is a central component of large-scale forest C monitoring and reporting practices prescribed by the Intergovernmental Panel on Climate Change (IPCC). Using a region–wide, repeated forest inventory, forest C stocks and stock change by pool were examined by LU categories. In eastern US forests, LU change is a substantial component of C sink strength (~37% of forest sink strength) only secondary to that of C accumulation in forests remaining forest where their comingling with other LUs does not substantially reduce sink strength. The strongest sinks of forest C were study areas not completely dominated by forests, even when there was some loss of forest to agriculture/settlement/other LUs. Long-term LU planning exercises and policy development that seeks to maintain and/or enhance regional C sinks should explicitly recognize the importance of maximizing non-forest to forest LU changes and not overlook management and conservation of forests located in landscapes not currently dominated by forests.

## The Growing Question of Land Use and the Forest Carbon Sink in the US

The global carbon (C) cycle has increasingly been the focus of monitoring efforts^1^ and policy discussions^2^ due to its role in the greenhouse gas effect and projected future climate change via anthropogenic C emissions, resulting in a concomitant increase in the monitoring of terrestrial C. As forests serve as the largest terrestrial sink of C^3^, the monitoring of land-use (LU) change (i.e., loss or gain of forests) and forest C dynamics (i.e., net C balance within forests remaining forest) are a critical component of national-scale reporting mechanisms under the United Nations Framework Convention on Climate Change (UNFCCC). Land-use change has been suggested to be the primary driver of the net emission of 156 Pg C globally between the years 1850 and 2000 with almost 60% occurring in tropical areas^4^. Given the importance of forest C and potential loss of the sink through deforestation, a component of the Intergovernmental Panel on Climate Change (IPCC) Good Practice Guidance is solely dedicated to Land Use, Land-Use Change and Forestry^1^. In prior UNFCCC submissions, the US has not provided complete estimates of changes in forest C stocks by afforestation, deforestation, and forests remaining forest (i.e., LU change accounting)^5^. Initial research^6^ suggests that LU is the dominant factor controlling rates of forest C accumulation in the US. Much of the current US forest C sink strength has been attributed to forest expansion and recovery resulting from substantial LU changes (urban, farm, and industrial establishment) from the late 1700 s to the early 1900 s^7^. The abandonment of farms, especially in the eastern US during the mid to late 1800 s^8^, coupled with the rise of modern forest management in the 1900 s (including afforestation of large areas in the southeastern US^9^), has been speculated as the primary driver of the forest C sink in the 1900 s to the present^7^,^10^,^11^. Results from forest ecosystem process models have strongly suggested that LU change, or more broadly disturbance history, is a major driver of the eastern US forest carbon sink^12^. More recently^13^ it was found in the southeastern US that net forest LUC contributed ~6 Tg C yr^−1^ to the annual C sink compared to net forest accumulation of ~75 Tg C yr^−1^. As identifying the influence of LU on the terrestrial C cycle in the US is not only suggested by IPCC Good Practice Guidance^1^ and UN reviews^5^ but is also critical to monitoring forest C sink strength, its examination is paramount. Under a business-as-usual future scenario it has been forecasted^14^ that perhaps 36% of the land area in the conterminous US will change LU between 2001–2051 which in turn will have profound implications for the US terrestrial C sink and potential US commitments to reductions in greenhouse gas emissions.

To quantify LU change effects on the forest C sink in the eastern US^15^ we used a systematic, repeated forest inventory^16^ of eastern US forest C pools and LUs to estimate forest C stock and stock change by classes of LU and LU change. In the eastern US the national forest inventory consists of ~170,000 sample locations where land use (not land cover) is interpreted from high resolution imagery (~1 m) and if a forest LU is present at the sample location field crews measure tree/site attributes for estimation of forest C pools. To examine this national inventory at spatial extents relevant to study objectives and appropriate for the inventory sampling intensity, LU (among three general classes: agriculture, forest, and settlements/other) and forest C attributes were summarized within individual 1,384 km^2^ hexagons which numbered over 3,000 for the study region. The LU and forest C attributes across study hexagons (i.e., landscapes) were considered individual study observations for the purposes of this investigation (see Methods).

## Forests and Settlements Gain at the Expense of Agriculture

**Figure 1:**
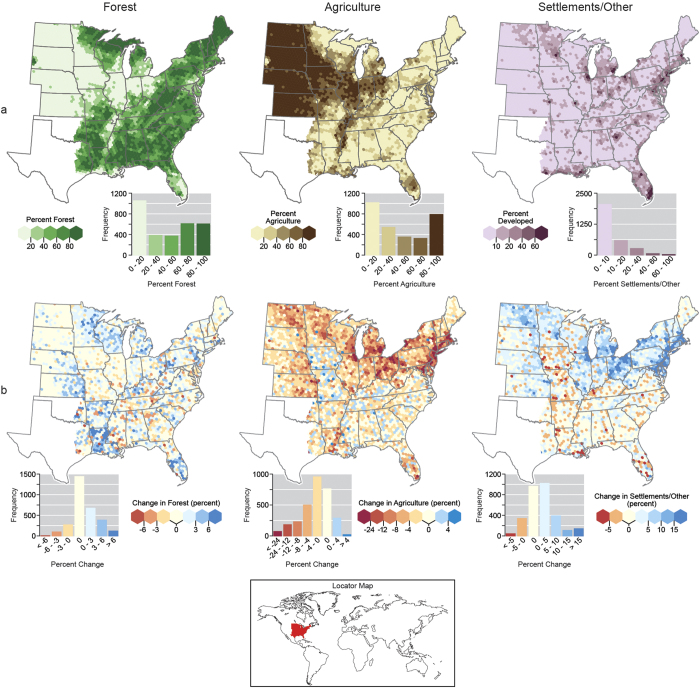
Percent (a) land use and (b) land use change by general land uses classes (forest, agriculture, and settlements/other) summarized for discrete hexagons in eastern US, 2001–2012. Maps generated using ArcGIS (www.esri.com/software/arcgis).

We found that forests are the dominant LU, exceeding 50%, across nearly half of the eastern US (Fig. 1a). Forest LU is highest, exceeding 80% of each hexagon’s area, in the upper Great Lakes, New England, along the Appalachian Mountain chain, and areas of the southeastern US. Agriculture was by the far the most dominant LU in the Great Plains extending into Illinois, Indiana, Ohio, and down the Mississippi River corridor. From 2001–2012, most hexagons had less than 5% change in forest LU (positive or negative) with gains in the southern Plains and Great Lakes states (Fig. 1b) and scattered losses in the central and south central US. In contrast, the agriculture LU experienced declines over much of the eastern US with the largest decreases across the Great Plains and northern states, especially in areas of southern New England. The settlements/other LU had strong gains (exceeding 15%) surrounding a number of metropolitan areas (e.g., Northeast Megalopolis) and across portions of select states (e.g., North Dakota, Ohio, and New York). Settlements/other LU changes appear to be opposite of agriculture: where agriculture has declined, settlements have increased^14^.

## Accompanying Pervasive Land-Use Change, Forest Carbon Stocks Increase

**Figure 2:**
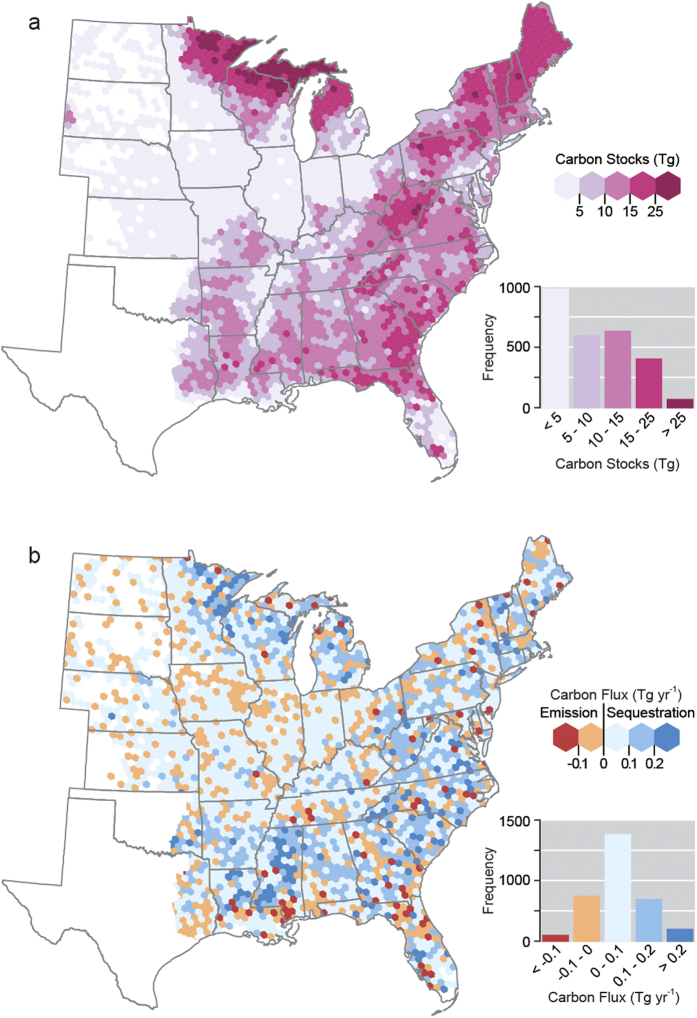
Forest land use (a) total ecosystem carbon stocks and (b) percent change in stocks by discrete hexagons, 2001–2012, eastern US. Maps generated using ArcGIS (www.esri.com/software/arcgis).

**Figure 3:**
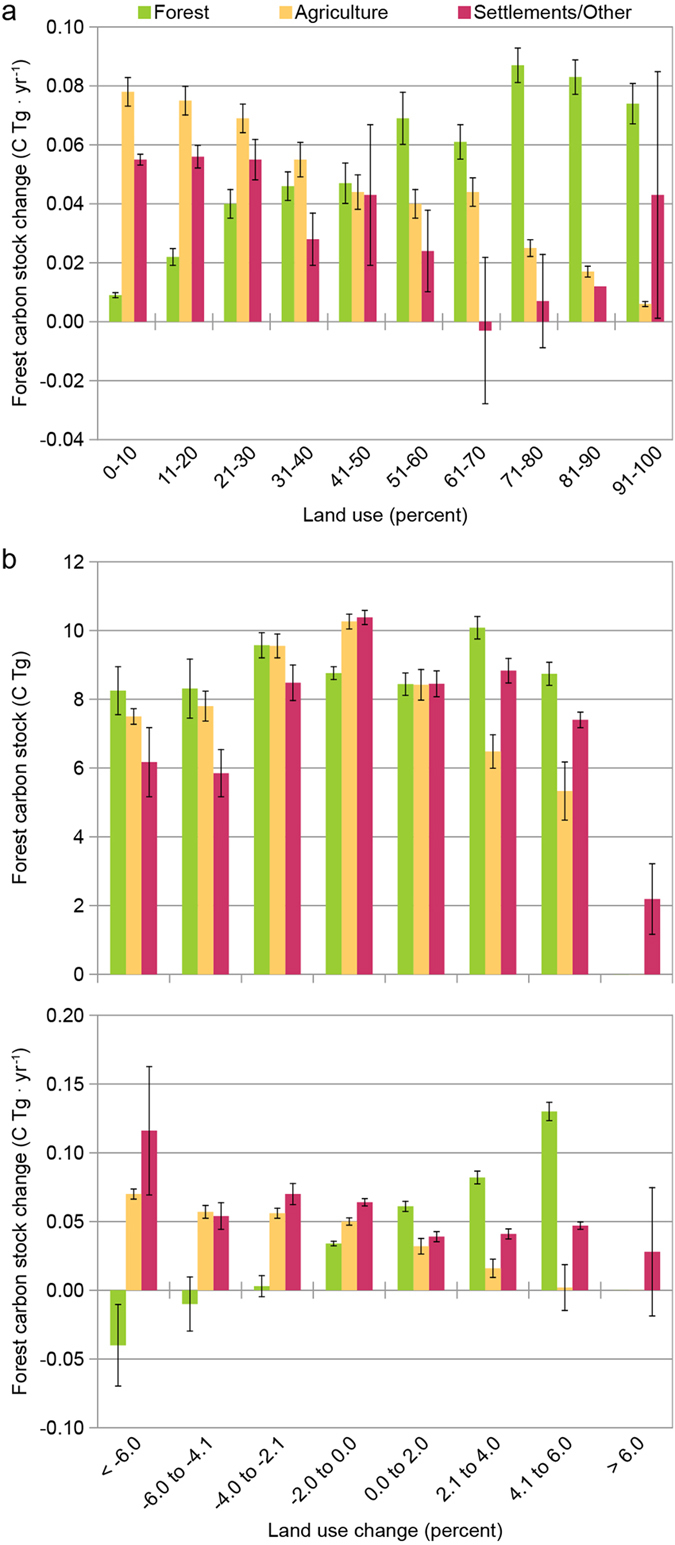
Forest carbon stock attributes by land use and land use change classes. (**a**) Mean stock change (Tg C yr^−1^) by classes of land use and (**b**) Mean stocks (C Tg) and stock change (Tg C yr^−1^) by classes of land use change, 2001–2012, eastern US (bar’s represent standard errors).

The spatial distribution of total forest ecosystem C stocks largely aligned with the distribution of the eastern US forest LU (Fig. 2a). The inclusion of pools such as litter and soil organic C resulted in mean stocks (Supplementary Table 1) being highest in northern MN and WI, New England, and coastal areas of the southeast. In terms of forest stock change, there was a skewing towards greater occurrence of increased C stocks in most regions of the US (Fig. 2b). The exception to this was the western Plains states including IA, MO, IL, and IN. An examination of mean annual changes in forest C stocks across LU classes indicated that the highest rates of net forest C accumulation were not in the most forested hexagons (Fig. 3a). Instead, the highest stock change rates were found in hexagons that had moderately high levels of forest LU (71–90%) and hexagons with minor amounts of agriculture (0–20%). The examination of forest C stocks and stock change by classes of LU change provided a complementary view (Fig. 3b). Mean stocks per hexagon were largely found to be above 8 Tg C for most categories of LU change although they appeared to be maximized in hexagons that had minimal changes in agriculture and settlements/other LUs. The lowest forest C stocks were found in hexagons that had the largest increase (>4%) in agriculture or settlements/other LUs. In terms of forest C stock change, forest C stocks increased in almost every LU change situation except when forest LU loss exceeded 6% (Fig. 3b). The largest increases (>0.1 Tg C yr^−1^) in forest C stocks were found in hexagons where settlements/other declined by over 6% and in hexagons where forest LU increased between 4.1 to 6.0%. Spearman rank-order correlations among the host of LU assessments and forest C stocks and stock change indicated that percent forest and agriculture LU were the most highly correlated with forest C stocks (coefficients = 0.94 and −0.89, respectively; *p < 0.05*) while percent forest and percent forest change were most highly correlated with stock change (coefficient = 0.32 and 0.38, respectively; *p* < 0.05) (Table 1).

## Strongest Forest Carbon Sinks are in Dynamic Landscapes

A strong eastern US forest C sink was ubiquitous despite the prevalence of non-forest LUs and associated LU changes. Interestingly, the strongest sinks of forest C were found in study hexagons not completely dominated by forests. Landscapes with 50–60% forest LU had statistically the same sink strength as landscapes with 90–100% forest. This finding may be associated with forests in highly productive regions where agriculture is a competitive LU to forests. These dynamics reflect the importance of accounting for LU change legacies and associated disturbances to forest ecosystems^12^, particularly given their documented importance in affecting forest composition, structure, and function at local and regional scales in the eastern US^8^,^12^,^17^,^18^. As the recovery of forests from disturbance or non-forest LU abandonment (i.e., old field succession) can often last decades or longer^8^,^18^, we hypothesize that the signal of long-term LU change legacies is reflected in our finding that the largest stocks and sequestration rates were in landscapes not completely dominated by forest. Using an ecosystem demography model, Albani *et al.*^12^ estimated that forests of eastern US had a net uptake of 140 to 250 Tg C yr^−1^ during the 1990’s which compares to approximately 145 Tg C yr^−1^ estimated in this study during the ~2000’s and suggests perhaps a weakening sink as hypothesized in other studies^12^,^13^. Furthermore, this result highlights the ability of forest ecosystems to recolonize non-forest LU’s which at some point in the past (perhaps centuries) may have been forest. It has been estimated^7^ that the settlement and subsequent deforestation and/or intensive harvest of US forests emitted over 4,000 Tg of C between 1715 and 1935. Certainly, most of that C emission cannot be recovered by contemporary eastern forests via LU transfers or forest growth. Although we would expect forest C sinks will become saturated^13^,^19^ once LU transfer into forests ceases combined with forest maturation; the strong sinks documented in comingled LU areas of privately owned lands^15^ suggests active LU planning and forest management as one opportunity to maintain C sink strength.

## Land Use Change is Second Largest Contributor to Forest Carbon Sink

**Figure 4:**
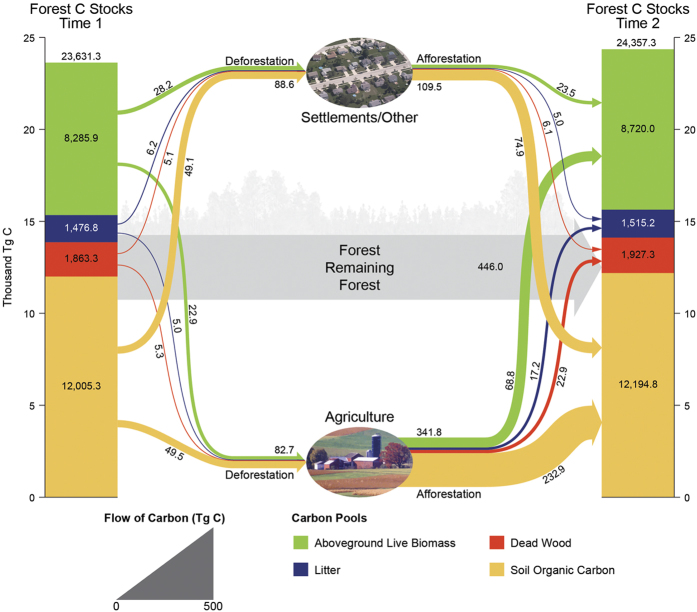
Forest carbon stocks and transfers (C Tg) by land use, land use change, and forest ecosystem pools, eastern US, 2001–2012, eastern US. (US Department of Agriculture Land Use depictions).

Forests remaining forest accrued nearly 446.0 Tg across the remeasurement period of 5 years (Fig. 4, Supplementary Table 1). In contrast, deforestation to the agriculture and settlements/other LUs resulted in a reduction of stocks by 82.7 and 88.6 Tg C, respectively. Afforestation of previous agriculture and settlements/other LUs resulted in an increase of forest C stocks by 341.8 and 109.5 Tg C, respectively. Hence, the effect of LU change on forest C stocks was a net increase of 280.0 Tg C (451.3 Tg C from afforestation minus 171.3 Tg C from deforestation) or 37% of the C accrual in forests remaining forest. In terms of forest C, our study found that only in the situation of substantial forest loss was there a concomitant loss in forest C stocks. While historical land-use patterns have been a driver of forest C sinks^8^,^12^,^17^,^18^, our results suggest this dynamic continues as contemporary LU change substantially contributes to the forest C sink in eastern US forests. The correlations between forest C stocks and agriculture LU (negative) and forest C stocks and percent of agriculture LU change (positive) (Table 1) reflect a period of ongoing agricultural LU abandonment and concomitant positive effects on forest C sinks. This LU dynamic can be further elucidated by examining the C transfers from agriculture/settlements/other LUs by individual pools as these were measured/modeled at time 2. The largest transfers of C into the forest LU were ordered as: agriculture soil organic C, settlements/other soil organic C, agriculture aboveground live biomass, settlements/other aboveground live biomass, agriculture litter, and agriculture deadwood. The transfer of C from settlements/other LUs should not be considered trivial as soil organic C and living biomass pools were larger than transfers from the minor agricultural pools of litter and deadwood. These results substantiate the ability of non-forest LUs to enhance terrestrial C sinks with policy implications for encouraging afforestation and working forests in these areas. Regardless of potential long-term saturation^19^ or continued sink maintenance^20^, the results of our study suggest that over a third of the eastern US forest C sink would be gone if transfers into the forest LU ceased.

## How Might We Increase the Forest Carbon Sink in the Eastern US?

Given the continually rising levels of atmospheric CO_2_, there may be a future where there are strong social movements to decarbonize economies and sequester as much C as possible^21^. Maintaining the forest LU via protection or expansion of wilderness areas is one policy consideration, but perhaps an additional approach may be to ensure maximum afforestation and forest growth within diverse LU landscapes. Explicitly acknowledging the effect of comingled LUs would greatly inform forest management and LU planning policies as the vast majority of eastern US forests are privately owned^4^. Moreover, providing incentives for maintaining and expanding forest use in agriculturally-dominated landscapes to enhance C sinks, will serve to reinforce other efforts focused on increasing the functionality of these areas from a biodiversity and ecosystem services perspective^22^. The effect of LU change on forest C stocks may be temporally sensitive with legacies of LU change (decades to centuries ago^12^) comingling with recent LU changes (~5 years as observed in this study) which in turn can obfuscate delineation of LU versus forest management effects on forest C. Therefore, forest and LU dynamics should be considered simultaneously when examining forest C dynamics and/or management across landscapes. Such a discipline of investigation may be considered complementary to traditional forest management sciences focused primarily on sustaining the forest C stocks (e.g., designing drought resilient stand structures and species mixtures to sustain forested areas under future global change). Indeed, even as the forest C sink (i.e., net C sequestration) may diminish in the future, tremendous stocks of forest C still exist that need to be maintained and which may be overlooked in policy discussions^23^. Perhaps it will not be the altered tree regeneration dynamics in response to climate change^24^ that will affect forests the most in the eastern US, but the migration of humans in response to climate change that may once again drastically alter the trajectory of forest C as seen with manifest destiny in the 1700 and 1800 s^7^.

## Methods

### Sample Design

Our study relies on a national forest inventory established and maintained by the USDA Forest Service Forest Inventory and Analysis (FIA) program, which is the primary source of information about the extent, condition, status, and trends of forest resources across the US^4^. The FIA program uses a nationally consistent sampling design covering all ownerships across the US^16^. A rotating panel repeated measure sample design is used and is based on a global sampling design^25^. Each panel and the entire sample are systematic across the US based on a triangular grid. The triangular grid is isotropic and each sample location represents a hexagonal area of 2,403 ha. One permanent sampling location was randomly chosen within each hexagon. It should be acknowledged that there are numerous sources of error^26^ to be considered when scaling up estimates of forest C such as sampling, measurement, and model selection. The systematic allocation of FIA’s sample points across space and time through a paneling system allows for statistically valid compilation at scales smaller than states^16^ as used in this study.

### Land Use Classification

Fine-scale remotely sensed imagery^27^ is partially used to define the LU at each sample location which has a nominal spatial resolution (raster cell size) of 1 m^2^. Prior to field measurement of each panel, each sample location in the panel is photo-interpreted manually to determine LU. For the purposes of this study, the breadth of various LU categories manually identified by photo interpreters was collapsed into three general LU categories: forest, agriculture, and settlements/other^28^ (Supplementary Table 4). Those sample locations determined to be a forest LU, potentially a forest LU, or where a forest LU was present at a previous measurement are verified by crews in the field to finalize LU identification. Sample locations determined to contain a forest LU have subsequent forest measurements collected.

### Forest Measurements

Only sample locations that are identified as a forest LU are measured for tree/site attributes in the field component of the inventory. Field inventory plots established in forested conditions consist of four, 7.32-m fixed-radius subplots spaced 36.6 m apart in a triangular arrangement with one subplot in the center^28^,^29^. All trees (live and standing dead) with a diameter at breast height (1.37 m) of at least 12.7 cm are inventoried on forested subplots. Within each sub-plot, a 2.07 m microplot offset 3.66 m from sub-plot center is established where only live trees with a diameter at breast height between 2.5 and 12.7 cm are inventoried.

### Data and Analysis

Field data for this study were taken entirely from the publicly available national FIA database^30^ using the forest inventory in 37 states of the eastern US (Fig. 1) for a total of 170,205 plots (all LUs) first established between 2002–2006 and remeasured 5-years later from 2007–2012. This study used a stock change approach as a surrogate for net C flux where the total stock of C by pool (e.g., aboveground live biomass) was estimated at two points in time with the difference divided by the remeasurement period (in years) serving as an estimate of average annual flux (C Mg ha^−1^∙yr^−1^). Forest ecosystem pools were delineated as: (1) aboveground live trees, (2) deadwood (including standing dead and downed dead), (3) litter, and (4) soil organic carbon. An ecosystem approach was used for C sink/source nomenclature where positive values indicate sequestration and negative values indicate an emission. For details regarding the estimation of each forest ecosystem C pool please see Supplementary Material.

To facilitate analysis at spatial scales suited for this study’s sampling intensity, forest C and LU was summarized by a hexagonal grid developed for this study (different from the hexagonal system used to allocate individual forest inventory plots). The use of discrete hexagons appropriately sized (1,384 km^2^) for the FIA plot network sampling intensity (median of 58 sample points per hexagon) facilitated visual interpretation of spatial patterns and evaluation of conclusions regarding forest C attributes in the context of LUs. As each hexagon was a considered an observation in this study, forest C stocks and LU change was estimated for each hexagon that had at least 8 sample points within it which excluded hexagons with a majority of their area out of population (e.g., Canada). A population estimate of total forest ecosystem stocks at time one and time two was computed in addition to stock and stock change for each C pool. The percentage of LU was calculated for each hexagon at each time based on the LU at the plot center of each sample point summed by each of the three LU categories divided by the total number of sample points in each hexagon. Land use change was calculated as the change in the percent LU between time one and time two by LU category.

Means and associated standard errors of forest C stocks and stock change were calculated for each LU class by 10% increments for both LU (time two) and change in LU across all study observations (i.e., hexagons). The spatial patterns in these results were evaluated visually through the choropleth mapping of LU (time two) and LU change, along with forest C stocks and stock change, by hexagons. Spearman rank-order correlation coefficients were calculated between forest C stocks and stock change versus LU (time two). Finally, in order to conceptualize the flow of forest C among pools and LUs across time, the population totals of forest C were determined for each hexagon then summed for the entire study region. Within each hexagon, C estimates for each FIA plot observation were weighted by the area it represented within the hexagon which was afforded by the systematic properties of the monitoring system^16^ (see Supplemental information).

## Additional Information

**How to cite this article**: Woodall, C. W. *et al.* Monitoring Network Confirms Land Use Change is a Substantial Component of the Forest Carbon Sink in the eastern United States. *Sci. Rep.* **5**, 17028; doi: 10.1038/srep17028 (2015).

## Supplementary Material

Supplementary Information

## Figures and Tables

**Table 1:** Spearman rank-order correlation coefficients between forest carbon stocks and stock change versus land use (time 2) and land use change metrics, eastern US (all *p* < 0.05 unless italicized).

Land Use and Land Use Change Metrics	Forest Carbon Stocks (C Tg)	Forest Carbon Stock Change (Tg C yr^−1^)
Percent forest	0.94	0.32
Percent forest change	*0.01*	0.38
Percent agriculture	−0.89	−0.29
Percent agriculture change	0.11	−0.13
Percent settlements/other	−0.14	*−0.03*
Percent settlements/other change	−0.12	−0.10
