# Supplementary material for: Monitoring Network Confirms Land Use Change is a Substantial Component of the Forest Carbon Sink in the eastern United States
Source: Sci Rep. 2015 Dec 7;5:17028. doi: 10.1038/srep17028 (PMC4671014; doi:10.1038/srep17028)
Supplement: Supplementary Information [file srep17028-s1.pdf]

Supporting Information

Manuscript: "Monitoring Network Confirms Land Use Change is a Substantial Component of the Forest Carbon Sink in the eastern United States"

Authors: Woodall, C.W., Walters, B.F., Coulston, J.W., D'Amato A.W., Domke, G.M., Russell, M.B., Sowers, P.A.

Table 1. Means and associated standard errors of total forest ecosystem carbon stocks (C Tg) by percent of land use at time 2 (forest, agricultural, and settlements/other) among study hexagons, 2002-2012, eastern US

Land Use Classes (percent)	Land Use					
	Forest		Agriculture		Settlements/other	
	Mean	Std Err	Mean	Std Err	Mean	Std Err
0-10	0.59	0.02	17.53	0.28	9.64	0.18
11-20	2.07	0.04	13.17	0.2	8.44	0.24
21-30	3.82	0.08	10.74	0.19	7.19	0.33
31-40	5.76	0.12	7.95	0.2	6.41	0.38
41-50	7.48	0.17	6.31	0.19	4.71	0.42
51-60	9.26	0.18	5.11	0.18	3.76	0.56
61-70	10.99	0.17	3.53	0.13	3.02	0.58
71-80	13.61	0.21	2.21	0.07	2.31	0.38
81-90	15.95	0.27	1.09	0.04	1.55	0.56
90-100	19.65	0.35	0.41	0.02	0.57	0.15

Table 2. Total area (km^2) and mean forest carbon stock density (numerator: total forest ecosystem stock for all pools, denominator: total area regardless of land use; Mg C ha^{-1}) by percent of land use at time 2 (forest, agricultural, and settlements/other) among study hexagons, 2002-2012, eastern US.

Land Use Classes (percent)	Land Use					
	Forest		Agriculture		Settlements/Other	
	Area	C Density	Area	C Density	Area	C Density
0-10	28,523	4.3	36,502	126.7	114,771	66.7
11-20	58,008	14.9	101,125	95.2	116,411	60.9
21-30	67,276	27.6	113,631	77.6	61,263	52.0
31-40	93,418	41.6	102,733	57.4	46,147	46.3
41-50	118,523	54.1	126,899	45.6	32,394	34.0
51-60	146,356	66.9	118,273	36.9	16,275	27.2
61-70	249,302	79.4	136,313	25.5	9,912	21.8
71-80	354,656	98.4	187,136	15.9	11,594	16.7
81-90	371,506	115.2	348,370	7.9	2,402	11.2
90-100	384,451	142.0	667,527	2.9	6,694	4.1

Methods: Estimation of Forest Ecosystem Carbon Pools

Standing dead and live tree C stocks were calculated in this study using the Component Ratio Method (CRM, Woodall et al. 2011). Briefly, the CRM facilitates calculation of tree component biomass (e.g., tops and limbs) as a proportion of the bole biomass based on component proportions from Jenkins et al. (2003). For standing dead trees, which may lack some or all of the components calculated using CRM (e.g., loss of limbs), structural and decay reduction factors were applied by decay class and species (Domke et al. 2011). The final step in C estimation was the conversion of standing dead/live total biomass to C mass assuming 50 percent C content of woody biomass. Belowground estimates of coarse root C were not included. The estimation of the soil organic carbon

(SOC), forest floor, and downed dead wood pools were accomplished using plot-level models used in the US' NGHGI (Smith et al. 2013, EPA 2014) where these carbon pools are based on models estimated using variables such as live-tree C density and stand age with coefficients by region and/or forest type.

Methods: Calculation of Confidence Intervals for Figure 4

The approximate 95 percent confidence intervals were formed by doubling the sampling errors which were computed by dividing the population total estimates into the square root of the variances (see Bechtold and Patterson 2005, page 59) using the SAS procedure SURVEYMEANS (varsum statement using the Taylor Series method; SAS Institute Inc. 201) for each estimate of forest C (Tg) in the diagram assuming simple random sampling (see Bechtold and Patterson 2005, page 25).

Table 3. Approximate 95 percent confidence intervals for categories of land use and land use change by forest carbon pool displayed in Figure 4 (Tg C) based on carbon population totals across Walter's hexagons included in this study

Component	Pool	Upper CI	Lower CI
Forest Stocks Time 1	Aboveground Live	8338.12	8233.88
	Dead Wood	1483.48	1468.52
	Litter	1876.27	1849.73
	Soil Organic Carbon	12076.83	11933.17
	Total	23742.50	23517.50
Forest Stocks Time 2	Aboveground Live	8765.30	8656.70
	Dead Wood	1522.75	1507.25
	Litter	1940.56	1913.44
	Soil Organic Carbon	12267.03	12122.97
	Total	24462.72	24233.28
Forest Remaining Forest	Total	481.21	410.79
Forest Flux to Agriculture	Aboveground Live	24.87	19.85
	Dead Wood	5.36	4.56
	Litter	5.76	4.78
	Soil Organic Carbon	53.73	45.37
	Total	86.77	77.51
Forest Flux to Settlements/Other	Aboveground Live	30.91	25.03
	Dead Wood	5.49	4.69
	Litter	6.75	5.61
	Soil Organic Carbon	53.02	45.26
	Total	94.34	82.42
Agriculture Flux to Forest	Aboveground Live	71.91	63.29
	Dead Wood	18.07	16.53
	Litter	24.08	21.72
	Soil Organic Carbon	242.53	223.27
	Total	352.34	329.06
Settlements/Other Flux to Forest	Aboveground Live	25.63	20.77
	Dead Wood	5.39	4.59
	Litter	6.63	5.57
	Soil Organic Carbon	81.93	67.79
	Total	116.11	102.19

Methods: Identification of Land Uses

Plots interpreted as non-forest are assigned a corresponding land use code at plot center: 1) agriculture land, 2) rangeland, 3) settlements, 4) other, 5) non-census water, and 6) census water (USDA 2014, pages 73-74) where non-census water bodies are typically too

small to be considered in the national census but are encountered at the scale of an FIA plot. Excluding the rangeland and water land use groups, all other groups have a number of specific, associated non-forest land use sub-classifications (e.g., cropland versus orchard under the agricultural land use major category). Non-census and census water bodies were excluded in this study. Because the "other" land use category only accounted for approximately 2.8 percent of study observations and was largely represented by land uses that could not support vegetation (e.g., barren rock or beaches), it was included in the settlements/other land use class. The response variables used in the Spearman's Rank Correlation were first evaluated for spatial autocorrelation using Moran's I and Geary's C (Cressie 1993). Test results suggested that there was very little spatial autocorrelation for the forest carbon stock and stock change estimates for the centroids of the hexagons used in the study. Based on these findings and the scale of the study area (entire eastern US), spatial autocorrelation was deemed to not affect results to a detectable level.

Table 4. Land use classification, definitions, and number of observations in this study, eastern US

Specific Land Use Category	Definition	Number of Study Sample Points
Forest Land	A land-use category that includes areas at least 36.6 m wide and 0.4 ha in size with at least 10 percent cover (or equivalent stocking) by live trees of any size, including land that formerly had such tree cover and that will be naturally or artificially regenerated.	73,497
Agricultural Land	Land managed for crops, pasture, or other agricultural use. Includes rangelands defined as areas composed of grasses, forbs, or shrubs. Includes other wetlands.	80,373

Settlements/Other	Land used primarily by humans for purposes other than forestry or agriculture. Also includes non-vegetated land uses such as beaches, rock, and ice.	16,335

References

Bechtold, W.A., Patterson, P.L. *The enhanced Forest Inventory and Analysis program—national sampling design and estimation procedures*. Gen. Tech. Rep. SRS-GTR-80. 85 pp. (U.S. Department of Agriculture, Forest Service, Northern Research Station, 2005).

Cressie, N.A. in *Statistics for spatial data, revised edition*. 928 pp (Wiley, 1993).

Domke, G.M., Woodall, C.W., Smith, J.E. 2011. Accounting for density reduction and structural loss in standing dead trees: Implications for forest biomass and carbon stock estimates in the United States. *Carbon Balance and Manag*. **6**, 14 (2011).

US EPA. *Forest sections of the Land Use, Land Use Change, and Forestry chapter, and Annex. In: US Environmental Protection Agency, Inventory of US Greenhouse Gas Emissions and Sinks: 1990-2012*. (United States Environmental Protection Agency, 2014). Available at: http://www.epa.gov/climatechange/ghgemissions/usinventoryreport.html. (Accessed: 13th October 2015).

Jenkins, J.C., Chojnacky, D.C., Heath, L.S., Birdsey, R.A. National scale biomass estimators for United States tree species. *Forest Sci*. **49**, 12-35 (2003)

SAS Institute Inc. *SAS/STAT(R) 9.3 user's guide*. (SAS Institute, Inc., 2011)

Smith, J.E., Heath, L.S., Hoover, C.M. Carbon factors and models for forest carbon estimates for the 2005–2011 National Greenhouse Gas Inventories of the United States. *For. Ecol. Manage*. **307**, 7-19 (2013).

USDA. Pages 73-74; Supplementary Table 3 in *Forest Inventory and Analysis national core field guide, Volume I: Field, data collection procedures for phase 2 plots, version 6.1*. (U.S. Department of Agriculture Forest Service, Forest Inventory and Analysis, 2014) Available at: http://www.fia.fs.fed.us/library/ (Accessed: 13th October 2015).

Woodall, C.W., Heath, L.S., Domke, G.M., Nichols, M.C. *Methods and equations for estimating volume, biomass, and carbon for trees in the U.S. forest inventory, 2010*. Gen. Tech. Rep. NRS-GTR-88, 30 pp (U.S. Department of Agriculture, Northern Research Station, 2011).
